# Low levels of hybridization between sympatric Arctic char (*Salvelinus alpinus*) and Dolly Varden char (*Salvelinus malma*) highlights their genetic distinctiveness and ecological segregation

**DOI:** 10.1002/ece3.1583

**Published:** 2015-07-07

**Authors:** Shannan L May-McNally, Thomas P Quinn, Eric B Taylor

**Affiliations:** 1 Department of Zoology, Biodiversity Research Centre and Beaty Biodiversity Museum, University of British Columbia6270 University Ave., Vancouver, BC, Canada, V6T 1Z4; 2 School of Aquatic and Fishery Sciences, University of WashingtonSeattle, Washington, 98195

**Keywords:** Ecological segregation, hybridization, microsatellites, *Salvelinus*, sympatric populations

## Abstract

Understanding the extent of interspecific hybridization and how ecological segregation may influence hybridization requires comprehensively sampling different habitats over a range of life history stages. Arctic char (*Salvelinus alpinus*) and Dolly Varden (*S. malma*) are recently diverged salmonid fishes that come into contact in several areas of the North Pacific where they occasionally hybridize. To better quantify the degree of hybridization and ecological segregation between these taxa, we sampled over 700 fish from multiple lake (littoral and profundal) and stream sites in two large, interconnected southwestern Alaskan lakes. Individuals were genotyped at 12 microsatellite markers, and genetic admixture (*Q*) values generated through Bayesian-based clustering revealed hybridization levels generally lower than reported in a previous study (<0.6% to 5% of samples classified as late-generation hybrids). Dolly Varden and Arctic char tended to make different use of stream habitats with the latter apparently abandoning streams for lake habitats after 2–3 years of age. Our results support the distinct biological species status of Dolly Varden and Arctic char and suggest that ecological segregation may be an important factor limiting opportunities for hybridization and/or the ecological performance of hybrid char.

## Introduction

Natural hybridization is a fundamental evolutionary process in the biology of plants and animals (Mayr 1963; Arnold 1992; DeMarais et al. 1992; Barton 2001). When different species or genetically distinct populations interbreed, a wide variety of phenomena can result, for example, the formation of hybrid zones of various kinds, adaptive radiation, and reinforcement of pre- and postmating reproductive barriers during speciation (Schluter 1996; Arnold 1997; Dowling and Secor 1997; Seehausen 2004; Aboim et al. 2010). Consequently, studying the level of hybridization, the structure of hybrid zones, and the viability of hybrids can assess the strength of isolation between evolutionarily young lineages and may signal factors relating to the origin and maintenance of species differences and the evolution of reproductive isolation (Barton and Hewitt 1989; Schluter 1996; Arnold 1997; Jiggins and Mallet 2000). Further, defining species boundaries from studies of contact zones can also be critical when designing management goals for morphologically cryptic or hybridized populations (Campton 1987; Allendorf et al. 2001; Bickford et al. 2007).

Natural hybridization is frequent in several vertebrate groups, especially fishes (Schwartz 1972; Campton 1987; Bernatchez et al. 1995; Allendorf and Waples 1996; Arnold 1997; Rieseberg 1997; Scribner et al. 2001). External fertilization (Hubbs 1955), niche overlap (van Herwerden and Doherty 2006) and competition for limited spawning sites (Campton and Utter 1985) have probably contributed to a high incidence of hybridization in fishes. For salmonid fishes (*Salmo*, *Oncorhynchus*, *Salvelinus*, and related genera), hybridization is common among particular pairs of species (reviewed in Taylor 2004). Many salmonids exhibit few or no intrinsic postzygotic barriers to interbreeding (e.g., genomic incompatibilities) and this may be one of many factors contributing to the high levels of hybridization (Taylor 2004). Consequently, salmonids are well suited to studies on prezygotic isolating barriers, such as habitat segregation, or extrinsic postzygotic barriers, such as ecologically dependent selection against hybrids (Redenbach and Taylor 2003; Taylor 2004; Rogers and Bernatchez 2006).

**Figure 1:**
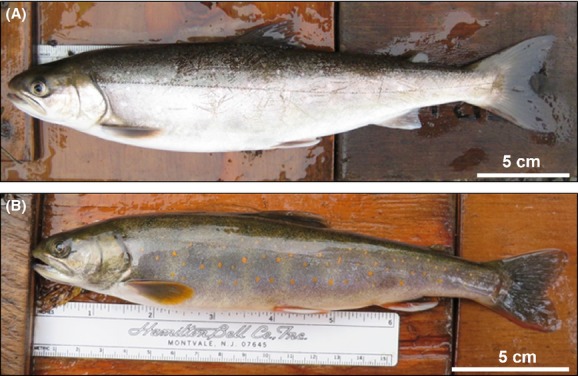
(A) Arctic char (*Salvelinus alpinus*) from Happy Creek, Lake Aleknagik, AK; note the silver body with white spots, narrow caudal peduncle and well-defined fork in the tail; (B) Dolly Varden (*Salvelinus malma*) from Yako Creek, Lake Aleknagik; note the small colorful orange spots along a darker green body, thick caudal peduncle, and paddle-shaped tail.

Among salmonids, char (*Salvelinus*) have proved instrumental in our understanding of evolution and speciation in fishes due to their diversity, polymorphism, and adaptability to different habitats (Snorrason et al. 1994; Gíslason et al. 1999; Brunner et al. 2001; Jonsson and Jonsson 2001; Klemetsen 2010; Reist et al. 2013). Of seven well-recognized species of char, the Arctic char (*Salvelinus alpinus,* Fig.1) may show the greatest variability in morphology, colouration, ecology, and genetic structure, leading to the naming of multiple taxa within the Arctic char “species complex” (Klemetsen et al. 2003; Klemetsen 2013; Reist et al. 2013). In fact, such diversity has contributed to some debate over the status of the Dolly Varden (*Salvelinus malma,* Fig.1) as a species distinct from Arctic char (McPhail 1961; Brunner et al. 2001; Taylor et al. 2008).

In the eastern Bering Sea and associated freshwater habitats, Arctic char and Dolly Varden are abundant in the western and southern portion of Alaska, USA, around the Alaska Peninsula, and along the northern slope adjacent to the Canadian western Arctic (Reist et al. 1997; Mecklenburg et al. 2002). Alaskan Arctic char are typically permanent residents in lakes, where they spawn on submerged beaches at sizes of 50 cm or more in total length. Dolly Varden char are more commonly anadromous, that is, spawning in streams and feeding by juveniles in streams before seaward migration where the majority of growth occurs before returning to streams to spawn at sizes typically also in excess of 50 cm. In addition, some Dolly Varden in western Alaska may reside permanently in streams and mature at sizes of 20 cm or less. Notwithstanding the basic lacustrine–anadromous life history differences between Arctic char and Dolly Varden, the two species may overlap spatially in streams as juveniles (e.g., DeLacy and Morton 1943; McPhail 1961). Although consistent morphological differences exist between Arctic char and Dolly Varden throughout this zone of sympatry (e.g., pyloric ceca, gill raker counts), McPhail (1961) identified populations that had ambiguous morphology and speculated that they resulted from hybridization between the species which is not uncommon in *Salvelinus* (e.g., Hammar et al. 1991; Taylor et al. 2001; Redenbach and Taylor 2003). Further, mitochondrial (mtDNA)-based surveys by Brunner et al. (2001) supported the idea of recent or ongoing hybridization between the species in the Bering Sea area, leading the authors to suggest that the separate species status of Dolly Varden was “questionable”. By contrast, Taylor et al. (2008) sampled several lakes in western Alaska and found that although the two species shared mtDNA haplotypes, they were very distinct at nine microsatellite loci, supporting their status as valid biological species. In one southwestern Alaskan lake (Lake Aleknagik), however, preliminary data suggested that about 7% of the fish sampled had ambiguous genetic identity and were tentatively classified as hybrids. A combination of relatively low sample size (∼60–100 in two lake–stream watersheds) and lack of sample site diversity (2–6 sites per watershed) in the Taylor et al. (2008) study limited their ability to accurately access the level of hybridization, that is, percentage of all fish classified as having hybrid genotypes, between Dolly Varden and Arctic char, especially among distinct habitats, and to what extent interspecific differences in habitat use might influence hybridization. Specifically, sampling fish over a range of ages and sizes from a variety of different habitats within stream and lake environments are needed to more accurately estimate the frequency of hybridization and the ecological processes that might permit, or hinder, the production of hybrids, their habitat use, and survival.

In this study, we report the results of extensive sampling of Arctic char and Dolly Varden in Lake Aleknagik and Lake Nerka, southwestern Alaska to assess the following: (1) the degree of genetic divergence and levels of hybridization between species, and (2) the degree of interspecific ecological segregation and ontogenetic habitat use patterns by parental species and their hybrids. The level of hybridization and differences in distribution between the species were assessed across a wide variety of habitats in streams and lakes to determine whether habitat structure influenced hybrid distribution and prevalence. Based on the preliminary knowledge of their ecology, we hypothesized that Dolly Varden would be found only in stream environments, Arctic char would be predominantly lacustrine, and the hybrids would be intermediate in habitat use, occupying the lower reaches of streams or lake shores. Determining the extent of hybridization and the ecological distribution of parental species and hybrids between Arctic char and Dolly Varden can contribute to our understanding of the role that ecology plays in the evolution and persistence of reproductive isolation between sympatric species.

## Materials and Methods

### Sample collection

**Figure 2:**
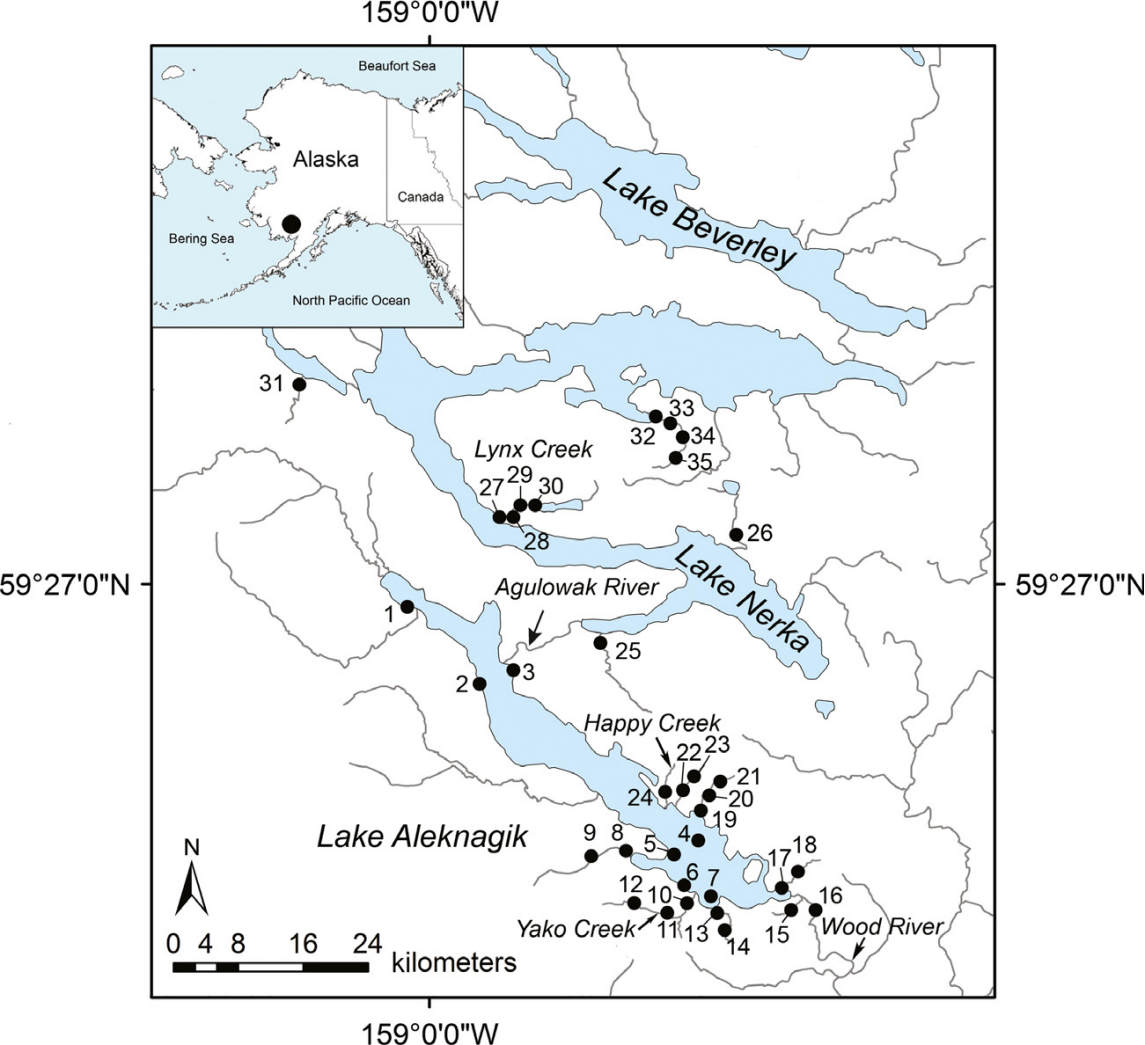
Map showing sampling localities in Lake Aleknagik and Lake Nerka, the first two interconnected lakes in the Wood River Lakes system, southwestern Alaska, USA. 1–7 = Aleknagik lake sites; 8–9 = Bear Creek; 10–12 = Yako Creek; 13–14 = Whitefish Creek; 15 = Silver Salmon Creek; 16 = Wood River; 17–18 = Mission Creek; 19–21 = Eagle Creek; 22–23 = Hansen Creek; 24 = Happy Creek; 25 = Fenno Creek; 26 = Teal Creek; 27–30 = Lynx Creek; 31 = Little Togiak River; 32–35 = Hidden Lake Creek.

Lake Aleknagik (83 km^2^, 32 km in length, maximum depth of 110 m, mean depth of 43 m) is located in the central portion of Bristol Bay and near Dillingham, southwestern Alaska (Fig.2). Lake Aleknagik is the lowermost lake in the Wood River system, a series of five interconnected lakes that drain via the Wood River into the Nushagak River to the south and eventually into Bristol Bay. The lakes are oligotrophic and range from 3 to 45 km in length (Burgner 1991). Lake Aleknagik is home to a diverse native fish community including pygmy whitefish (*Prosopium coulterii*), three-spine sticklebacks (*Gasterosteus aculeatus*), slimy and coastrange sculpins (*Cottus cognatus* and *C. aleuticus*), and salmonids (Salmonidae), with char and sockeye salmon (*Oncorhynchus nerka*) being the most numerically dominant salmonids. Upstream of Lake Aleknagik is Lake Nerka (45 km in length, 201 km^2^, maximum depth of 164 m, mean depth of 39 m), which discharges into Lake Aleknagik via the Agulowak River. This river is short (6.4 km) and readily passable by migratory fishes.

Char were sampled during July and August 2012 and 2013 from 24 sites in Lake Aleknagik (*N *=* *496, Fig.2, Tables1 and S1) representing seven sites in the lake and 17 riverine sites using a combination of sinking gillnets, beach seines, stick seines, minnow traps, and angling. The adipose fin was removed from each fish and stored in 95% ethanol for laboratory analysis. Fish classified as young-of-the-year (YOY) based on body size were euthanized in a dilute solution of MS-222 (tricaine methanesulfonate) and stored whole in 95% ethanol. Fork length (mm) and Global Positioning System (GPS) coordinates of the sampling site were recorded for each fish collected. For lake sites 4, 5, and 7, sinking gillnets were set by boat daily for 4–6 h and suspended above the bottom with floats. These sites were selected to represent various depths and included a shallow (Site 7, ∼5–15 m deep), intermediate (Site 4, ∼15–20 m deep), and deep (Site 5, ∼20─50 m deep) site. At each of these sites, two gillnets were used to sample different size classes of char. Both nets were 30 m long by 2 m high, but one had 30-mm and the other had 20-mm stretched mesh. Beach sites (Sites 2, 3, 6) were selected from various regions around the lake and were sampled using a 32 m long × 5 m deep beach seine net tapered from 5 m deep in the centre 6-m-wide section with 6-mm mesh to 13-mm mesh in the 2.5-m side panels and 1 m deep with 30-mm mesh at the edge panels. It was deployed to shore during the day by boat in a semi-circle reaching approximately 10–15 m offshore. Most of the samples from streams were collected using a 2.5-m-long stick seine with a mesh size of approximately 5 × 5 mm. Minnow traps baited with salmon eggs were also used in Hansen Creek sites 22–23.

**Table 1:** Summary of sampling locations, date, total number of fish genotyped (*N*), and proportion of samples assigned as Arctic char (*S. alpinus*) (AC), Dolly Varden (*S. malma*) (DV), and putative hybrids (HYB) in the Wood River Lake system, southwest Alaska. Arctic char were defined as having *Q*_DV_ values of ≤0.21, Dolly Varden were defined as having *Q*_DV_ ≥ 0.78, and hybrids were defined as having *Q*_DV_ values between 0.21 and 0.78

	Latitude and longitude	Date	*N*	Total AC	Total DV	Total HYB
Lake Aleknagik	59.343301°N-158.806686°W	July–September 2012	324	295	27	2
		July–August 2013	164	77	87	0
Lake Nerka	59.559679°N -159.023666°W	June–August 2012	189	166	22	1
		July–August 2013	33	17	16	0
Wood River	59.262936°N -158.573313°W	September 2012	8	0	7	1

Samples of adult (maturing) Arctic char and Dolly Varden from Lake Aleknagik and the Wood River were collected in September, 2012 by angling. The adult Arctic char were caught off the mouth of Youth Creek (Site 1), on the north side of Lake Aleknagik, and the adult Dolly Varden were sampled from the Wood River which drains Lake Aleknagik into Bristol Bay to the south (Site 16). In addition to the samples from Lake Aleknagik and its tributaries, samples were also collected from 11 riverine sites in Lake Nerka (*N *=* *222) by stick seining and angling (Fig.2, Tables1 and S1). In total, tissue samples from 718 Arctic char and Dolly Varden were collected at 35 sites across lakes Aleknagik and Nerka.

### Microsatellite analysis

Tissue samples were digested, and total genomic DNA was extracted using the DNeasy DNA blood and tissue extraction kit (Qiagen Inc., Valencia, CA, USA). Extracted DNA samples were stored at −20°C for use in multiplex polymerase chain reactions (PCR) using the Qiagen multiplex kit (Cat. No. 206145). DNA was amplified in 10 *μ*L PCRs at 95°C for 15 min, 94°C for 30 sec, 35 cycles of 1.5 min at an annealing temperature of 55°C followed by 72°C for 1 min, and 60°C for 30 min. Microsatellite variation was assayed using primers labeled with fluorophores and a 3730S 48-capillary DNA Analyzer with GS 500 LIZ or 600 LIZ internal size standards (Applied Biosystems, Carlsbad, California, USA). Alleles were manually scored using the program GeneMapper (GeneMapper v.3.7, Applied BioSystems). We amplified 13 microsatellite loci isolated from other salmonid species: Atlantic salmon, *Salmo salar* (*SSOSL456*; Slettan et al. 1997); bull trout, *Salvelinus confluentus* (*Sco200, Sco202, Sco215, Sco216, Sco220*; DeHaan and Ardren 2005); chinook salmon, *Oncorhynchus tshawytscha* (*OtsG83b, OstG253b*; Williamson et al. 2002); Dolly Varden, *Salvelinus malma* (*Smm-17, Smm-21, Smm-22, Smm-24*; Crane et al. 2004); and rainbow trout, *Oncorhynchus mykiss* (*OMM1105*; Rexroad et al. 2002) (see May-McNally 2014 for full details).

### Statistical analysis of microsatellite DNA

We used MICRO-CHECKER (van Oosterhout et al. 2004) to check the microsatellite data for errors in scoring such as stuttering and null alleles which can compromise subsequent analyses. Next, we used FSTAT ver 2.9.3 (Goudet 2001) and ARLEQUIN ver 3.5 (Excoffier and Lischer 2010) to generate the basic descriptive statistics of sample size (*N*), number of alleles (*NA*), allelic richness (*AR*), and observed (*HO*) and expected (*HE*) heterozygosity. Tests for departures from Hardy–Weinberg equilibrium (HWE) for each locus–locality combination were performed with GENEPOP ver 4.2 (Raymond and Rousset 1995) using an exact test in which probability values were determined using a Markov chain method (*P*) controlling for multiple tests as per Narum (2006).

To obtain a general assessment of the number and distinction of genetic groups in Lake Aleknagik and Lake Nerka without a priori assignment to taxon, we conducted a factorial correspondence analysis (FCA) in GENETIX (Belkhir et al. 2001) on allele frequencies across all loci of Lake Aleknagik and Lake Nerka char alone, and with reference populations of allopatric Arctic char from the central Canadian Arctic (*N *=* *43) and a reference sample Dolly Varden (*N *=* *29) from the Egegik fishing district located south of Dillingham and Bristol Bay, Alaska, at the outflow of Becharof Lake (see Taylor et al. 2008 and Hart et al. 2015 for details). Learning samples of Arctic char and Dolly Varden were assigned to species using a combination of morphology, biogeography, and diagnostic microsatellite markers (see below). The FCA is a multiallelic factor analysis that summarizes variation in categorical variables (allele counts across multiple loci) in allelic space across a small number of dimensions. To test for population subdivision for all samples pooled within Lake Aleknagik and Lake Nerka, we ran simulations of *K = *1 to *K = *10, repeated five times in the Bayesian program STRUCTURE ver. 2.3.4 (Pritchard et al. 2000) for each sampling year (2012, 2013) separately to detect any interannual differences that might exist and were the data using DISTRUCT (Rosenberg 2004). STRUCTURE is a model-based clustering algorithm that assigns individuals to a *K* number of genetic groups that minimizes departures from HWE and LD within populations based on their multilocus genotypes. To infer support for the most probable number of subpopulations for each sampling year, Δ*K* (Evanno et al. 2005) was calculated across multiple runs of STRUCTURE using STRUCTURE HARVESTER (Earl and von Holdt 2012).

To assign individuals as Arctic char or Dolly Varden after confirming that a *K *=* *2 was an appropriate model (see above and Results), we used an *ad hoc* approximation of species identity where fish whose *Q* values were ≥ 0.95 (i.e., at least 95% of the genome characteristic of Dolly Varden, *Q*_DV_) were classified as Dolly Varden and fish whose *Q*_DV_ values were ≤ 0.05 were classified as Arctic char. Preliminary assignment of *Q* value groups to species was based on established morphological differences between Arctic char and Dolly Varden (e.g., see McPhail 1961). Any fish that had *Q*_DV_ values ≥ 0.05, but ≤ 0.95 were noted as possible hybrids. Next, we used a simulation approach to generate a range of *Q*_DV_ values that would be indicative of hybrids (see Vähä and Primmer 2006). First, we used the program HYBRIDLAB (Nielsen et al. 2006) to create 200 simulated F_1_, F_2_ and backcrossed hybrids generated from the random selection of alleles from each locus in the reference population of Arctic char from the central Canadian Arctic (same as above) and in Dolly Varden sampled from Vancouver Island and the Queen Charlotte Islands in British Columbia (BC, *N *=* *40). We used BC Dolly Varden from northern BC because we were unsure of the exact river of origin of the Egegik Dolly Varden and whether or not they were truly allopatric relative to Arctic char. The Dolly Varden from BC are a different subspecies (*S. malma lordi*) than the Egegik Dolly Varden (*S. m. malma*, see review by Kowalchuk et al. 2010; Taylor and May-McNally 2015), but the results of simulations using either subspecies did not change and we gained the advantage of knowing that the BC Dolly Varden are allopatric with respect to Arctic char. Next, these reference populations were designated as “learning samples” by implementing the USEPOPINFO model combined with simulated hybrids and char from Lake Aleknagik and Lake Nerka through five replicated analyses in STRUCTURE (Pritchard et al. 2000). We took the average values between the upper and lower limits of possible *Q*_DV_ values for the simulated hybrids across the five replicated analyses to create a “zone of hybridity”. Admixed individuals from the Wood River basin whose *Q*_DV_ values fell inside this range were classified as putative hybrids and were used to generate the level of hybridization for each lake. Our sample sizes for allopatric Arctic char and Dolly Varden used in the simulation above, especially for Arctic char, were relatively modest. Consequently, we generated a second simulated zone of hybridity using 50 Arctic char from Lake Aleknagik and Lake Nerka with *Q*_DV_ values closest to 0 (*Q*_DV_ < 0.05, pure Arctic char) and 50 Dolly Varden with *Q*_DV_ values closest to 1.0 (*Q*_DV_ ≥ 0.88, pure Dolly Varden) using the same methods as above. Both zones of hybridity were then compared to the range generated by Taylor et al. (2008) for Arctic char and Dolly Varden in southwestern Alaska. The STRUCTURE analyses were conducted using a burn-in period of 50,000 Markov Chain Monte-Carlo (MCMC) iterations proceeded by an additional 450,000 steps, replicated five times to verify consistency across runs.

We also used NEWHYBRIDS vers.1.1 Beta 3 (Anderson and Thompson 2002) as an alternative procedure to identify parental species and hybrids. NEWHYBRIDS is also a model-based approach, but uses the multilocus genotypes to assign individual fish to one of six genotypic classes: parental Arctic char, parental Dolly Varden, F_1_ hybrids, F_2_ hybrids, backcrosses to Arctic char or backcrosses to Dolly Varden. An estimate of the posterior probability of belonging to each of the six genotype classes was obtained for each individual fish and assigned to that class for which this probability was highest. The same allopatric populations used in the STRUCTURE analysis were also run in NEWHYBRIDS among the samples as nonadmixed reference populations for a minimum of 200,000 MCMC steps under the uniform priors option.

### Spatial distribution across sample sites

Genotyped samples of Arctic char, Dolly Varden, and hybrids were positioned spatially by using their GPS coordinates to map the distribution of the species among and within streams. Species assignment was based upon admixture (*Q*_DV_) values, and fish that were classified as hybrids (see above) were noted. The GPS coordinates of sampling sites were mapped using ArcGIS 10.2.2 (ESRI, Redlands, CA, USA). Variation in spatial distribution was assessed by analyzing sampling sites and sampling years separately. To determine the distribution of age classes between lake and stream sites for each char species in Lake Aleknagik, a length-frequency histogram was generated for fish of each species at each sampling site. Histogram shape has been used effectively to divide individuals into age classes in other salmonid fishes (see Matthews et al. 1997), and the five modes we resolved corresponded approximately to age categories of 0+ to >4+ for Wood River Lake system char.

## Results

### Microsatellite variability

MICRO-CHECKER did not find evidence of scoring errors or to large allele dropout in any of the samples. Evidence for a nonamplifying (“null”) allele was found in some samples for locus (*Sco216*) and this marker was therefore removed. When samples were partitioned into Dolly Varden and Arctic char (see below), all loci were polymorphic except for *Smm-21* in Arctic char, which was monomorphic and diagnostic for each species; all Arctic char were homozygous for a 110 base pair allele. By contrast, Dolly Varden were polymorphic at *Smm-21* with alleles that ranged in size from 120 to 132 (Table S1). Arctic char and Dolly Varden had similar numbers of alleles (17.8 vs 17.3) when pooled across all sites. Dolly Varden showed the highest number of alleles for a single locus (39 at locus *OtsG83b* followed by 38 at locus *Sco200*). When examining samples pooled across sites within each lake and for Arctic char and Dolly Varden separately (separation of species being based on STRUCTURE results, seen below), 17 of 46 tests for deviations from Hardy–Weinberg equilibrium (HWE; two lakes x 12 loci for Arctic char plus two lakes x 11 loci for Dolly Varden) were significant at *P *≤* *0.011. The majority of deviations from HWE originated within Dolly Varden with 13 of the 17 deviations found in lakes Aleknagik and Nerka Dolly Varden.

### Population structure

The FCA using known allopatric populations of Arctic char and Dolly Varden and the examination of the diagnostic locus *Smm-21* indicated that there were two groups of fish both in Lake Aleknagik and Lake Nerka in 2012 that were similar to the reference populations of Arctic char and Dolly Varden from western Alaska and the Canadian Arctic (Fig. S1). The numerically dominant genetic cluster (79% of samples) in Lake Aleknagik was taken to represent Arctic char as those individuals were homozygous at the diagnostic Arctic char locus (*Smm-21*) and grouped with reference populations of Arctic char in the FCA projection. The second cluster was numerically smaller (21%) and grouped with reference populations of Dolly Varden populations in the same FCA projection. Substantial differentiation as measured by pairwise F_ST_ was observed between the two species in Lake Aleknagik (F_ST_* *=* *0.172) and in Lake Nerka (F_ST_* *=* *0.186; *P *<* *0.001, Table2).

**Table 2:** Pairwise F_ST_ (*θ*) estimated by variation across 12 microsatellite DNA loci in sympatric Arctic char (*S. alpinus*) and Dolly Varden (*S. malma*) from Lake Aleknagik and Lake Nerka, Alaska and allopatric populations of Arctic char and Dolly Varden from the Canadian Arctic and Egegik, Alaska. Arctic char were defined as having *Q*_DV_ values of ≤ 0.21, Dolly Varden were defined as having *Q*_DV_ ≥ 0.78, and hybrids were defined as having *Q*_DV_ values between 0.21 and 0.78

	DV (Aleknagik)	DV (Nerka)	AC (Aleknagik)	AC (Nerka)	AC (Canadian Arctic)
DV (Nerka)	0.009^*^	–	–	–	–
AC (Aleknagik)	0.172	0.187	–	–	–
AC (Nerka)	0.172	0.186	0.007^*^	–	–
AC (Canadian Arctic)	0.169	0.179	0.124	0.128	–
DV (Egegik)	0.022	0.0163	0.156	0.149	0.181

AC, Arctic char, DV, Dolly Varden. Values accompanied by asterisks are not significantly >0 (*P* ≤ 0.0151; after adjustment for multiple simultaneous tests incorporating the false discovery rate procedure of Narum 2006).

**Figure 3:**
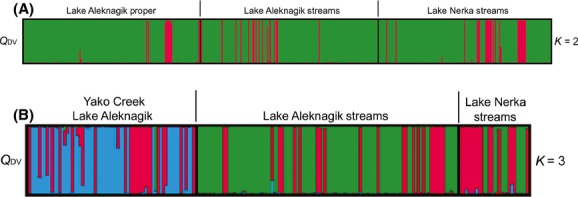
Results of STRUCTURE analysis for sympatric Alaskan Arctic char (*Salvelinus alpinus*) and Dolly Varden (*S. malma*) assayed at 12 microsatellite DNA loci and sampled from (A) Lake Aleknagik proper, streams and Lake Nerka stream sites in 2012 for *K *=* *2 (B) Yako Creek, Lake Aleknagik, Lake Aleknagik stream sites and Lake Nerka stream sites in 2013 for *K *=* *3. Color codes are: green* *=* *Arctic char, red* *=* *Dolly Varden, blue* *=* *additional population of Dolly Varden as defined by admixture (*Q*) values.

Analysis by STRUCTURE indicated that the most likely number of genetic populations across both lake and stream sites for both lakes in 2012 was *K *=* *2 (Fig.3A), as evidenced by a mean Δ*K* of 1675 vs. 1.1 for *K *=* *3 as the next most likely model. When sampling effort in stream habitats was increased in 2013, a *K *=* *3 model was best supported by the Δ*K* method as evidenced by a mean Δ*K* likelihood of 14.6 vs. 3.5 for *K *=* *5 as the next most likely model (Fig.3B). In the 2013 samples, the Δ*K *=* *3 analysis also supported a single population of Arctic char, but a two-population model for Dolly Varden. Specifically, within Lake Aleknagik, the Yako Creek sample appeared to contain two populations of Dolly Varden. In all analyses, including when species were run separately, only a single population of Arctic char was ever supported either within or between the two lakes.

For the initial *ad hoc* assignment of individuals to species across 2012 and 2013 samples, 558 fish (78% of samples) had *Q*_DV_ values ≤ 0.05 and were classified as “pure” Arctic char, whereas other 144 fish (20%) had *Q*_DV_ values ≥ 0.95 and were classified as “pure” Dolly Varden. All individuals classified as Arctic char by this method were homozygous for the diagnostic Arctic char locus *Smm-21* (allele 110). Likewise, all individuals classified as Dolly Varden by this method were polymorphic at this same locus (ranging from 120 to 132) and did not possess the diagnostic Arctic char 110 allele. A further 16 fish (2%) fell in between the range of 0.05 ≥ *Q*_DV_ ≤ 0.95 suggesting the number of hybrids was low (see below). Of these 16 fish, six had the alleles diagnostic of Arctic char and *Q*_DV_ values ranging from 0.072 to 0.216 whereas the other 10 were polymorphic for alleles diagnostic for Dolly Varden at the *Smm-21* locus and had *Q*_DV_ values ranging from 0.493 to 0.939.

### Genetic classification and hybridization levels

**Figure 4:**
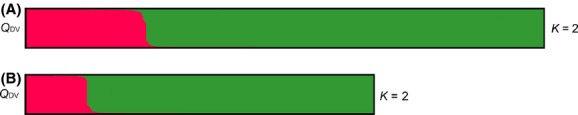
Overall genetic classification of 718 sympatric Alaskan Arctic char (*S. alpinus*) and Dolly Varden (*S. malma*) using 12 microsatellite loci from (A) Lake Aleknagik and (B) Lake Nerka. Assignment in STRUCTURE was based on the probability of belonging to two a priori genetic groups (*K *=* *2). Individuals are arranged according to decreasing *Q*_DV_ -values (i.e., the proportion of red in each line) and both sampling years, 2012 and 2013, are shown. Colour codes are as follows: red* *=* S. malma*, green* *=* S. alpinus*.

Analysis of the dataset with STRUCTURE with *K* set to 2 further suggested a general absence of hybridization between the two discrete genetic clusters representing Arctic char and Dolly Varden individuals, respectively (Fig.4A,B). Simulated hybrids were generated from allopatric populations of Canadian Arctic char and Dolly Varden and also from char sampled from Lake Aleknagik and Lake Nerka with admixture values indicative of pure species (see above). These simulated hybrids had *Q*_DV_ values that were ≤ 0.78 and ≥ 0.21 (using fully allopatric learning samples) and *Q*_DV_ ≤ 0.79 and ≥ 0.28, respectively (Lake Aleknagik and Lake Nerka learning samples, Fig S2). As both ranges were comparable, we used the zone of hybridity derived from the allopatric reference sample simulated hybrids (*Q*_DV_ between 0.78 and 0.21, average* *=* *0.44) in the subsequent analyses because it was most similar to the range generated by Taylor et al. (2008) for western Alaskan char (*Q*_DV_ between 0.78 and 0.23).

The range of *Q*_DV_ values for “pure” Arctic char (expressed as *Q*_DV_) averaged 0.0049 (*Q*_DV_ range 0.128–0.002), while the resultant admixture value for “pure” Dolly Varden averaged 0.99 (range 0.812 - 0.998). Using these ranges of *Q*_DV_ for parental and hybrid genotypes, STRUCTURE found that the majority of fish fell within the parental ranges of Arctic char and Dolly Varden and only three fish (0.60%) from Lake Aleknagik and one fish (0.45%) from Lake Nerka had admixture values within the zone of hybridity (Table1). These values did not change when samples were analyzed separately based on shared sampling year and site (e.g., same stream or lake site), and because of small numbers of putative hybrids, any tests of microhabitat differences affecting hybridization prevalence and occurrence were pursued no further. The four putative hybrids originated from Happy Creek, Site 5 (the deepest lake site in Lake Aleknagik), the Wood River main stem, and the uppermost stream site in Lynx Creek, Lake Nerka, and all were found in the 2012 samples.

Bayesian assignment of individuals into parental or hybrid (i.e., first- or late-generation hybrids or backcrosses) genotypes with NEWHYBRIDS indicated a higher proportion of hybrids than estimated by STRUCTURE for Lake Aleknagik (5% vs. 0.6% by STRUCTURE) and Lake Nerka (5% vs. 0.45%). A total of 77% (382 fish) of the Lake Aleknagik samples were classified as parental Arctic char, 18% (89) as parental Dolly Varden, 0% (0) as F_1_, 1% (5) as F_2_, 0% (0) as backcrosses with Arctic char and 4% (20) as backcrosses with Dolly Varden using NEWHYBRIDS (Table S3). The fish assigned as backcrosses with Dolly Varden were scattered across stream habitats in Lake Aleknagik, but with most of the hybrids were found in Yako Creek and Happy Creek. The F_2_ individuals originated from Happy Creek, upper Bear Creek (Site 9), and the Wood River. With the exception of one fish from Happy Creek, all F_2_ individuals had variable *Smm-21* alleles indicative of Dolly Varden. For Lake Nerka, 83% (184 fish) of samples classified as parental Arctic char, 12% (27) as parental Dolly Varden, 0% (0) as F_1_, 0% (0) as F_2_, 0% (0) as backcrosses with Arctic char and 5% (11) as backcrosses with Dolly Varden (Table S3). The fish assigned as backcrosses with Dolly Varden were found primarily in upper Lynx Creek.

### Spatial discreteness between species

Arctic char were found both in the littoral and offshore habitats of Lake Aleknagik and also in some stream habitats, whereas Dolly Varden were found only in streams (Fig. S3─7). In streams, Arctic char typically occupied sites near the mouth, but they were also present farther upstream, as much as several kilometers from the lake. Dolly Varden were generally found in the middle to upper reaches of most streams.

**Figure 5:**
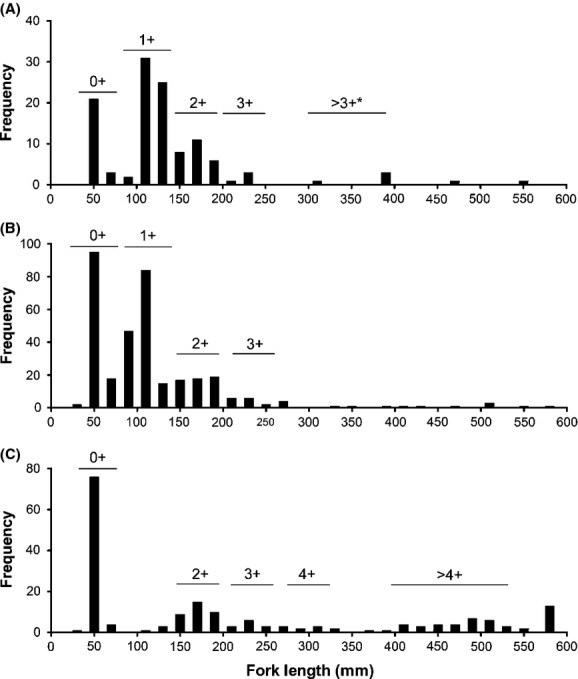
Size frequency histogram displaying estimated age classes of Arctic char (*S. alpinus*) and Dolly Varden (*S. malma*) from Lake Aleknagik (A) Dolly Varden from stream habitats with the exception of >3+ starred individuals collected from the Wood River in the autumn (B) Arctic char from stream habitats (C) Arctic char from lake habitats. Age 0 represents young-of-the-year fish, and “+” represents all older age classes combined. Arctic char were defined as having *Q*_DV_ values of ≤ 0.21, and Dolly Varden were defined as having *Q*_DV_ ≥ 0.78, while hybrids with *Q*_DV_ values between 0.21 and 0.78 were excluded.

The distribution of the different age classes across stream and lake habitats differed for Arctic char and Dolly Varden in Lake Aleknagik (Fig.5). Young-of-the-year (YOY) to mature ages classes of Dolly Varden were seen in stream habitats (Fig.5), but were not collected from any lake sites. Age 3+ Dolly Varden appeared to reach a maximum size of approximately 270 mm fork length and displayed vibrant spotting patterns suggesting maturing stream-resident fish (e.g., see Fig. 1). Young-of-the-year to age 2+ Arctic char were also present in streams, but fish >250 mm (3+ and older fish) were very rare in streams (Fig.5). In the lake, Arctic char were present at beach sites only as YOY. Age 1+ fish were virtually absent from any lake sampling, but 2+ and older fish were collected with gillnets set deeper in the lake, including much larger fish than were ever sampled in streams (Fig.5).

## Discussion

### Hybridization between Arctic char and Dolly Varden

The taxonomic relationship between Arctic char and Dolly Varden has been contentious owing to their morphological similarity, the documentation of hybrids between them, and the general lack of comparative ecological studies especially in sympatry (McPhail 1961; Brunner et al. 2001; Taylor et al. 2008; but see DeLacy and Morton 1943). Our data provide the most robust genetic analysis to date of the degree to which the two taxa are genetically distinct in sympatry and provide some preliminary indications concerning the ecological processes that maintain the apparently high level of genetic distinction and low level of hybridization between Arctic char and Dolly Varden.

First, the FCA projection showed that Arctic char and Dolly Varden sampled from two lakes in the Wood River Lake system clustered closely with their respective reference, allopatric populations of each taxon, and separately from one another. Moreover, the FCA projection identified few, if any, fish that were intermediate between the two clusters and that might represent hybrids. Finally, analyses in STRUCTURE indicated two highly distinct genetic clusters identified as Arctic char and Dolly Varden, and STRUCTURE and NEWHYBRIDS both suggested that hybrids between the species were comparatively rare.

Our data analyzed using STRUCTURE indicated that the level of hybridization between Arctic char and Dolly Varden in lakes Aleknagik and Nerka of the Wood River Lake system is minimal (0.6%, averaged over all sites) and much lower than the 7% level reported by Taylor et al. (2008) using the same analysis. The lower level of hybridization reported in our study compared with Taylor et al. (2008) could be a result of increased sample size (*N* = 718 vs. 105), additional microsatellite DNA markers used (12 loci vs. 9), a greater range of habitats sampled (27 discrete stream sites and various lake sites vs. six sites total), and more intensive upstream to downstream sampling within streams in our study which should have better represented the genetic diversity in the Wood River Lake system. The difference observed in the level of hybridization between the two studies could also result from the 7- to 10-year time span between studies as temporal fluctuations in environmental conditions across years can influence hybridization levels or the fitness of hybrids (Arnold 1997; Chenuil et al. 2000).

Subsequent analyses with NEWHYBRIDS supported the presence of late-generation hybrids; however, the proportion of F_1_ and F_2_ generation in lakes Alegnagik (0.0 and 0.01, respectively) and Nerka (both 0.0) was still very low. Low hybridization levels between the species in the Wood River basin appear comparable to other nearby systems containing sympatric populations of Arctic char and Dolly Varden. For example, in the Iliamna Lake system (also in Bristol Bay), Taylor et al. (2008) also found no evidence of F_1_ hybrids between the species, but classified approximately 10 of 65 individuals (15%) through admixture (*Q*) values as post-F_1_ hybrids. Our estimates should be more reliable overall, however, because Taylor et al. (2008) sampled only two discrete habitats from Iliamna Lake, a series of spring-fed ponds near Pedro Bay village comprised exclusively of small-bodied Dolly Varden, and a beach site in Iliamna Lake proper that had Arctic char. Given that Iliamna Lake is over 2600 km^2^, and only a small proportion of the genetic diversity of char was previously analyzed by Taylor et al. (2008), a genetic survey across the wider range of habitats present within the Iliamna Lake system may produce similar results to that seen in the Wood River Lake system (i.e., about 5% post-F_1_ hybrids). Finally, Gharrett et al. (1991) reported a high level of genetic distinction at allozyme loci between sympatric Arctic char and Dolly Varden from the Karluk River system, Kodiak Island, Alaska, and what appeared to be a low level of introgression between the species.

The NEWHYBRIDS analysis suggested that more hybrids (all post-F_1_) were found at certain stream habitats (e.g., Yako, Happy, and Lynx creeks) than at other sampling sites, but this is likely the result of generally larger samples sizes for these creeks. Because sampling effort was kept as consistent as possible within and across all streams, the larger sample sizes at these sites likely reflects an higher overall abundance of char in these habitats.

The STRUCTURE analyses did not find evidence of F_1_ hybrid individuals at any age class, and this result was supported through analyses in NEWHYBRIDS. In general, hybrid zones between Dolly Varden and bull trout also showed fewer F_1_ hybrids relative to other genotypic classes (e.g., Redenbach and Taylor 2003). In contrast to the STRUCTURE results, NEWHYBRIDS suggested that post-F_1_ hybrids may constitute up to 5% of the Lake Aleknagik population and that backcrossed hybrids are biased toward introgression with Dolly Varden. The NEWHYBRIDS algorithm is designed more to estimate the proportion of distinct hybrid classes present in a sample than STRUCTURE; therefore, it will tend to detect second- or third-generation hybrids that were classified as parental species when using *Q* threshold values employed in the STRUCTURE analysis (Vähä and Primmer 2006). The credible regions and error margins associated with each sample are accounted for differently in each program, so some of the potential late-generation hybrids with borderline *Q*_DV_ values nearing parental levels could have easily been misclassified as parental genotypes by the STRUCTURE analysis. The presence of advanced generation hybrids as suggested by NEWHYBRIDS analyses could explain the considerable overlap in morphology between species (e.g., gill rakers, pyloric ceca) in Lake Aleknagik, in contrast to the greater morphological separation of Arctic char and Dolly Varden in Iliamna Lake (McPhail 1961; Taylor et al. 2008).

### Factors influencing hybridization levels

When compared to other sympatric char systems where hybridization is often a regular occurrence such as Arctic char x lake char (*S. namaycush*: Hammar et al. 1989), Arctic char x brook trout (*S. fontinalis*: Hammar et al. 1991; Gross et al. 2004), Dolly Varden x bull trout (Baxter et al. 1997; Redenbach and Taylor 2003), the level of hybridization between Arctic char and Dolly Varden in the Wood River Lake system was low, especially considering their sister species status. In particular, our data indicated that F_1_ hybrids are generated rarely in this system, but the presence of some post-F_1_ individuals revealed that those that are produced are viable and fertile. Several ecological factors may constrain the opportunities for hybridization between Arctic char and Dolly Varden and maintain their genetic distinctiveness in the face of gene flow.

First, while other sympatric char systems provide evidence of spatial overlap of species in streams during breeding (e.g., bull trout and Dolly Varden, Bustard and Royea 1995; Hagen and Taylor 2001), Arctic char and Dolly Varden appear to use distinct spawning habitats. In the Wood River Lake system, Arctic char spawning sites are at the mouths of creeks on large submerged gravel beaches, whereas spawning Dolly Varden have only been observed in the Wood River itself and within streams such as Yako Creek (McBride 1980; C. Schwanke, Alaska Dept. of Fish and Game, Dillingham, AK, pers. comm.). Further, DeLacy and Morton (1943) reported that in the Karluk Lake system, Kodiak Island in the Gulf of Alaska, Dolly Varden were anadromous and spawned in streams and Arctic char were lake-dwelling and spawned in the lake. Different habitats used for spawning would clearly reduce opportunities for hybridization. Reported differences in spawning time probably also increase isolation (cf. Hagen and Taylor 2001); Dolly Varden have been observed spawning around the third week of September while Arctic char spawn in mid- to late October in the Aleknagik system (C. Schwanke, Alaska Dept. of Fish and Game, Dillingham, AK, pers. comm.).

Second, the apparent rarity of Dolly Varden in lakes Aleknagik and Nerka may also contribute to the low observed levels of hybridization. That is, the level of hybridization is to some extent dependent on the relative density of parental species (see Wirtz 1999). Across the habitats surveyed, Dolly Varden were numerically under-represented in comparison with Arctic char as seen by the ∼78% of samples genotyped that were classified as Arctic char. Even in streams, only about 30% of the samples were identified as Dolly Varden. The lower abundance of Dolly Varden in this system probably limits interspecific encounters during reproductive periods and thus may influence both the extent of hybridization and the directionality of introgression (Hubbs 1955; Mayr 1963; Taylor 2004; Burgess et al. 2005; Lepais et al. 2009).

Third, even when hybrids do form, postzygotic, environmentally dependent selection against hybrids may be an important factor constraining gene flow in the Arctic char – Dolly Varden system. Studies of hybrid zones have suggested that phenotypically intermediate hybrid individuals often perform poorly in parental niches (e.g., Hatfield and Schluter 1999; Godoy-Herrera et al. 2005), and DeLacy and Morton (1943) indicated that the two species have very different habitat use and migratory life history in sympatry (see above). Further, preliminary data from Lake Aleknagik suggest that Dolly Varden are primarily anadromous and Arctic char are nonanadromous and that the two species have different feeding habitats and associated morphological traits in strict sympatry (Dennert, A.M., May-McNally, S.L., Quinn, T.P., and E.B. Taylor unpubl. data).

Arctic char were the only species that we encountered during lake sampling, with younger (age 0+) age classes inhabiting littoral habitats and older individuals collected from deeper water (see also DeLacy and Morton 1943). From our mapping of genotypes by age class across habitats, it appears that age 0+ Arctic char enter streams from the lake to feed for some time, likely a year or more in some cases. Although these young age classes of Arctic char might have been under-represented in the gill nets and beach seine collections, it is also possible that they move into the small streams to exploit invertebrate prey while they are still small enough to occupy these small streams. Our data suggest that once certain size thresholds are met (estimated to be around ages 2+ or 3+), Arctic char appear to exit the streams and move back into the lake where they occupy deep water until maturity although they may make transient forays into streams again when large numbers of spawning sockeye salmon enter streams during late July through August where they prey heavily on salmon eggs (Eastman 1996; May-McNally 2014; Dennert, A.M., May-McNally, S.L., Quinn, T.P., and E.B. Taylor et al., unpubl. data). By contrast, our data further suggested that Dolly Varden, from ages 0+ to maturing fish, are only found in stream habitats (see also DeLacy and Morton 1943). Consequently, if the different habitat use apparently exhibited by juvenile Arctic char and Dolly Varden represents adaptation to alternative niches, and if hybrids between the two species are phenotypically intermediate relative to the parental species (as morphological data suggest – see McPhail 1961), then they may be at a disadvantage in parental niches and experience reduced survival or reproductive success. Such ecologically based postzygotic selection against hybrids may contribute to maintaining a high degree of genetic distinctiveness in the face of some gene flow as observed in many other systems (reviewed by Schluter 2001).

Alternatively, hybrids between Arctic char and Dolly Varden could exhibit equal or higher fitness than parental species in currently unknown, and presumably less common, intermediate habitats (Arnold 1997; Fritsche and Kaltz 2000; Moore et al. 2010; Culumber et al. 2011). Further, if the density of hybrids is low, as our data suggest, and selection against hybrids is frequency dependent, an evolutionary stable situation may develop where a low, but persistent level of gene flow occurs (Arnold 1997). This situation perhaps best fits the conceptual framework of Arnold’s (1997) “Evolutionary Novelty” model which posits that evolutionary stable hybrid lineages can arise under either environment-dependent or environment-independent selection and when hybrids can exhibit equivalent or higher fitness than parental genotypes, especially in ecotones or disturbed habitats (Anderson 1948; Moore 1977).

The spatial segregation and differences in life history between Dolly Varden and Arctic char described here mirror the distribution observed with other sympatric char such as Dolly Varden and bull trout. In northwestern British Columbia where they occur in sympatry, Dolly Varden are apparently limited to stream habitats, whereas bull trout are adfluvial; they spatially overlap with Dolly Varden in many tributaries as juveniles in their first year or two of life but migrate to lakes as older juveniles to feed before returning to streams to spawn as adults (Hagen and Taylor 2001). Although bull trout and Dolly Varden do not appear to be adapted to alternative trophic or habitat resources while juveniles are sympatric in streams (Hagen and Taylor 2001), the extent of any such differentiation between Arctic char and Dolly Varden, or adaptation to different spawning habitats (i.e., lakes versus streams), is unknown, but could contribute to resource-driven ecological segregation. The parallels in the interspecific differences in habitat use and life history and their possible roles in constraining gene flow in these two cases of sympatric char further suggest the role of ecology in the evolution, or at least the maintenance, of reproductive isolation (c.f., Hubbs 1961; Schluter and McPhail 1992; Smith and Skúlason 1996; Bernatchez et al. 1999; Hagen and Taylor 2001).

## Supporting Information

**Figure S1.** Factorial correspondence analysis (FCA) based on variation at 12 microsatellite DNA loci in sympatric Dolly Varden (DV) and Arctic char (AC) from Lake Aleknagik (DV = open black squares, AC = open blue squares) and Lake Nerka (DV = closed black squares, AC = closed blue squares) contrasted with respective reference populations of DV from the Egegik fishing district on the southwestern coast of Alaska (closed back triangles), and AC from Resolute Bay, Nunavut, in the Canadian Arctic (closed blue triangles). **Figure S2.** STRUCTURE plot generated from (A) Reference populations of Arctic char (*N = *43) from the Canadian Arctic and Dolly Varden (DV, *N = *40) from British Columbia, Canada with associated simulated hybrids (*N* = 50 each hybrid type) (B) Non-admixed Arctic char (AC, *N = *50) and Dolly Varden (DV, *N = *50) from the Wood River Lake system in southwestern Alaska and associated simulated hybrids (SH, *N = *150). **Figure S3.** Proportion of genotypes defined by admixture (*Q*_DV_) values corresponding to Arctic char (*Salvelinus alpinus*) and Dolly Varden (*S. malma*) sampled from within the lake proper and on beaches sites in Lake Aleknagik during summer 2012 and the Wood River during fall 2012 and assayed at 12 microsatellite DNA loci. **Figure S4.** Proportion of genotypes defined by admixture (*Q*_DV_) values corresponding to Arctic char (*Salvelinus alpinus*) and Dolly Varden (*S. malma*) sampled from stream sites in Lake Aleknagik during summer 2012 and assayed at 12 microsatellite DNA loci. **Figure S5.** Proportion of genotypes defined by admixture (*Q*_DV_) values corresponding to Arctic char (*Salvelinus alpinus*) and Dolly Varden (*S. malma*) sampled from stream sites in Lake Nerka during summer 2012 and assayed at 12 microsatellite DNA loci. **Figure S6.** Proportion of genotypes defined by admixture (*Q*_DV_) values corresponding to Arctic char (*Salvelinus alpinus*) and Dolly Varden (*S. malma*) sampled from stream sites in Lake Aleknagik during summer 2013 and assayed at 12 microsatellite DNA loci. **Figure S7.** Proportion of genotypes defined by admixture (*Q*_DV_) values corresponding to Arctic char (*Salvelinus alpinus*) and Dolly Varden (*S. malma*) sampled from stream sites in Lake Nerka during summer 2013 and assayed at 12 microsatellite DNA loci.

**Table S1.** Genetic diversity values at 13 microsatelllite loci for Arctic char (*Salvelinus alpinus*) and Dolly Varden (*S. malma*) from two southwestern Alaskan Lakes with two sampling years (2012-2013) pooled. **Table S2.** Lake Aleknagik and Lake Nerka site ID numbers and names with corresponding sampling locale, year(s) sampled, sample size (*N*) and given proportions of samples classified as Arctic char (*Salvelinus alpinus*) (AC), Dolly Varden (*S. malma*) (DV) and hybrid (HYB). **Table S3.** Summary of mean posterior probabilities with probability value ranges for individuals assigned with NEWHYBRIDS to each of six genotypic classes.
