# Supplementary material for: Low levels of hybridization between sympatric Arctic char (Salvelinus alpinus) and Dolly Varden char (Salvelinus malma) highlights their genetic distinctiveness and ecological segregation
Source: Ecol Evol. 2015 Jul 7;5(15):3031–45. doi: 10.1002/ece3.1583 (PMC4559047; doi:10.1002/ece3.1583)
Supplement: Supplementary file 1 [file ece30005-3031-sd1.docx]

AC

DV

**A**

**A**

DV

AC

**AC**

**DV**


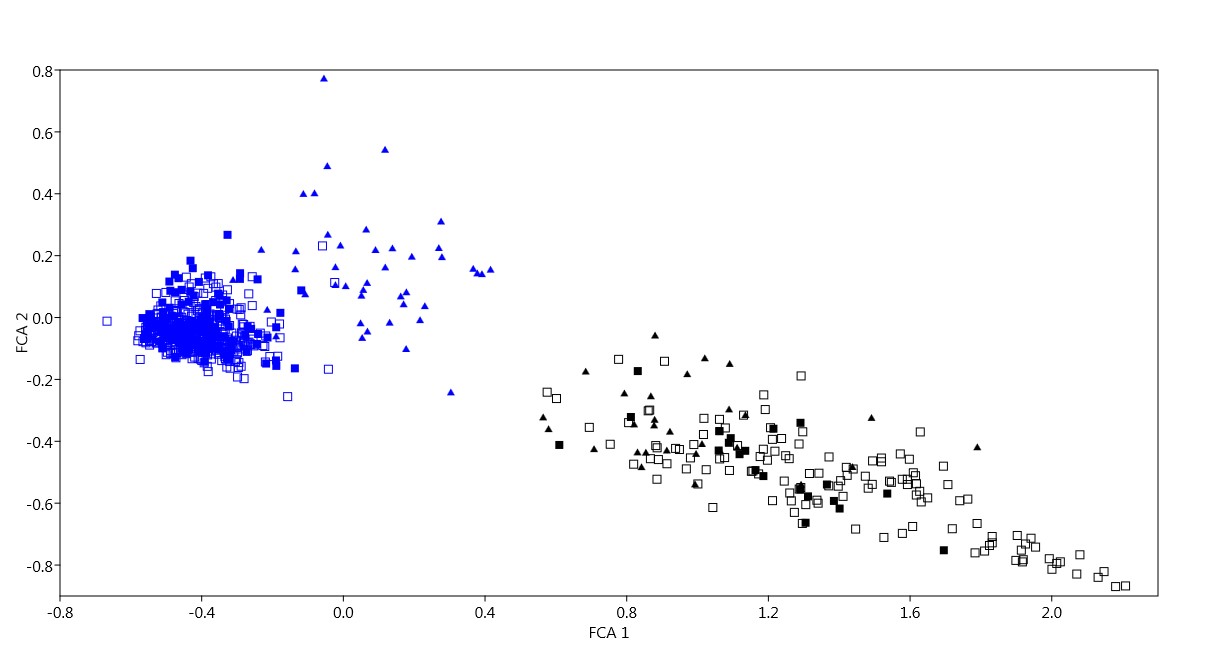


**Figure S1.** Factorial correspondence analysis (FCA) based on variation at 12 microsatellite DNA loci in sympatric Dolly Varden (DV) and Arctic char (AC) from Lake Aleknagik (DV = open black squares, AC = open blue squares) and Lake Nerka (DV = closed black squares, AC = closed blue squares) contrasted with respective reference populations of DV from the Egegik fishing district on the southwestern coast of Alaska (closed back triangles), and AC from Resolute Bay, Nunavut, in the Canadian Arctic (closed blue triangles).


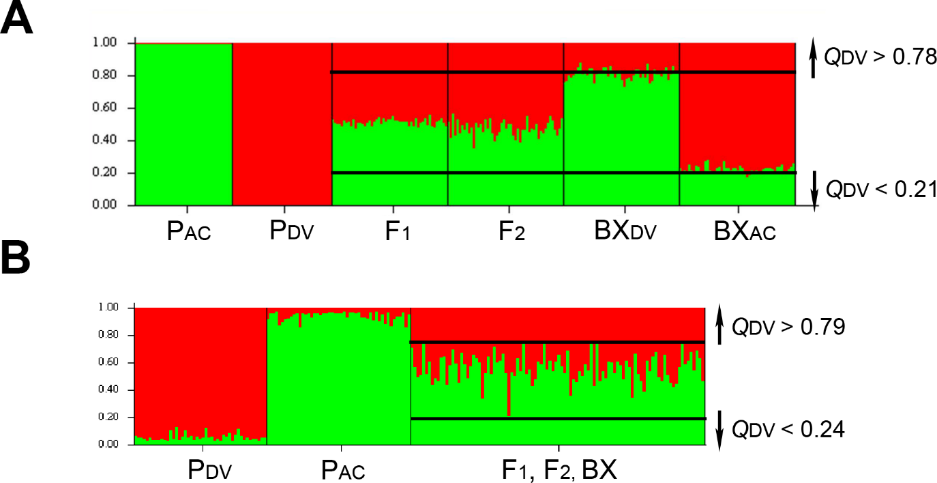


**Figure S2.** STRUCTURE plot generated from **(A)** Reference populations of Arctic char (*N =* 43) from the Canadian Arctic and Dolly Varden (DV, *N =* 40) from British Columbia, Canada with associated simulated hybrids (*N*= 50 each hybrid type) **(B)** Non-admixed Arctic char (AC, *N* = 50) and Dolly Varden (DV, *N* = 50) from the Wood River Lake system in southwestern Alaska and associated simulated hybrids (SH, *N* = 150). Horizontal bars indicate the upper and lower in the simulated hybrid plot represent the upper and lower boundaries for *Q*-value defined hybrids. P, parental generation, F1 and F2, first and second filial generations respectively, AC, Arctic char, DV, Dolly Varden, BX, backcrosses.

*

*


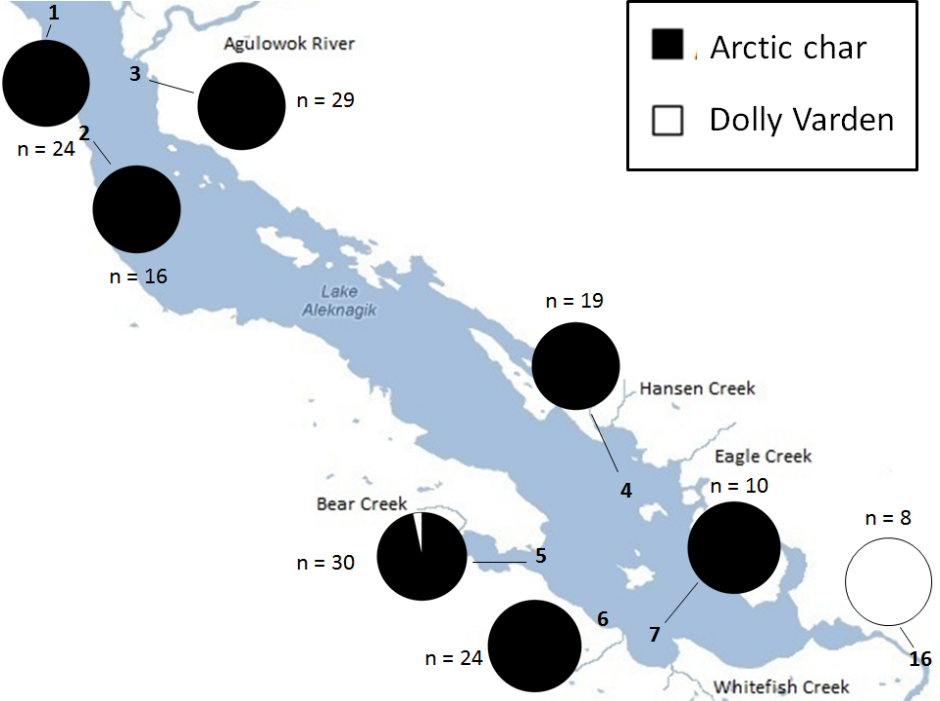


**Figure S3.** Proportion of genotypes defined by admixture (*Q*DV) values corresponding to Arctic char (*Salvelinus alpinus*) and Dolly Varden (*S. malma*) sampled from within the lake proper and on beaches sites in Lake Aleknagik during summer 2012 and the Wood River during fall 2012 and assayed at 12 microsatellite DNA loci. Arctic char were defined as having *Q*DV values of ≤ 0.21, Dolly Varden were defined as having *Q*DV ≥ 0.78, and hybrids were defined as having *Q*DV values between 0.21 and 0.78. A putative hybrid was found at each of the starred sites.

*


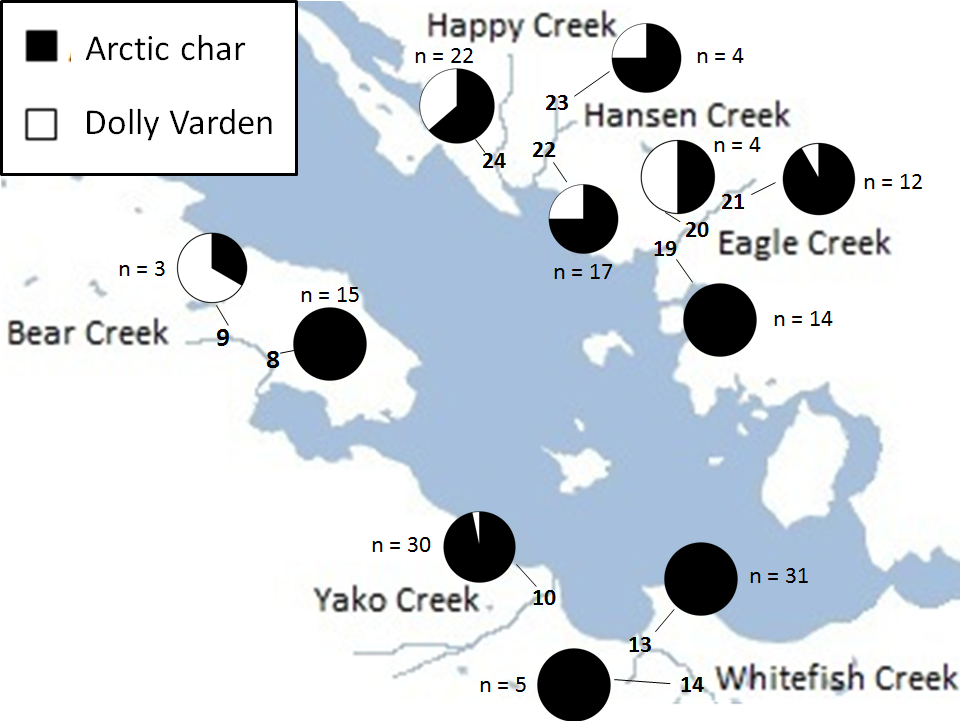


**Figure S4.** Proportion of genotypes defined by admixture (*Q*DV) values corresponding to Arctic char (*Salvelinus alpinus*) and Dolly Varden (*S. malma*) sampled from stream sites in Lake Aleknagik during summer 2012 and assayed at 12 microsatellite DNA loci. Arctic char were defined as having *Q*DV values of ≤ 0.21, Dolly Varden were defined as having *Q*DV ≥ 0.78, and hybrids were defined as having *Q*DV values between 0.21 and 0.78. A putative hybrid was found at the starred site.

*


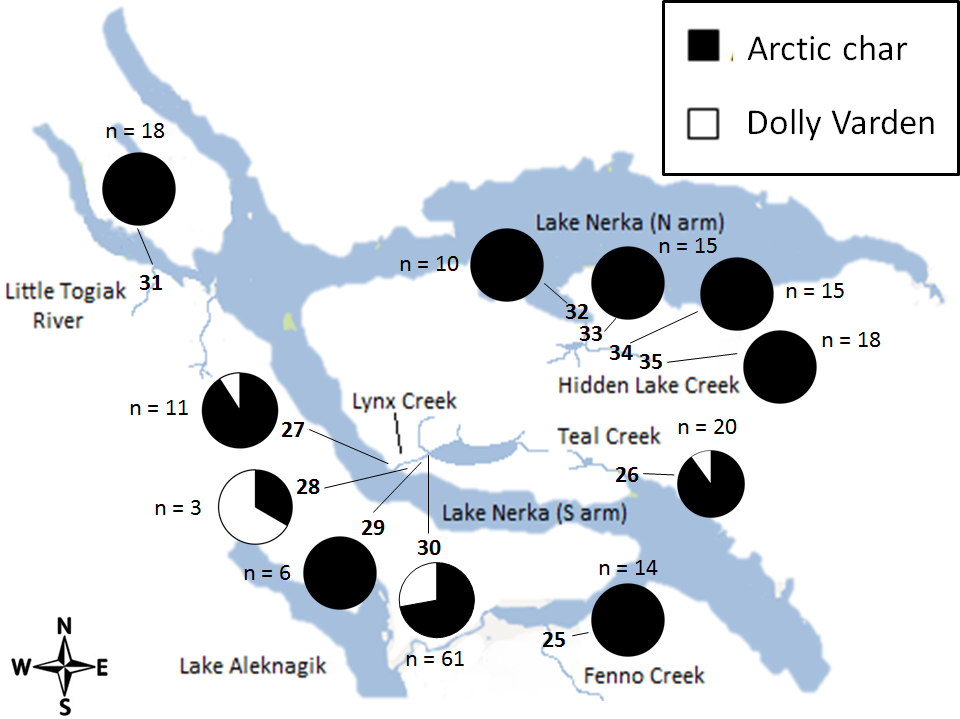


**Figure S5.** Proportion of genotypes defined by admixture (*Q*DV) values corresponding to Arctic char (*Salvelinus alpinus*) and Dolly Varden (*S. malma*) sampled from stream sites in Lake Nerka during summer 2012 and assayed at 12 microsatellite DNA loci. Arctic char were defined as having *Q*DV values of ≤ 0.21, Dolly Varden were defined as having *Q*DV ≥ 0.78, and hybrids were defined as having *Q*DV values between 0.21 and 0.78. A putative hybrid was found at the starred site.


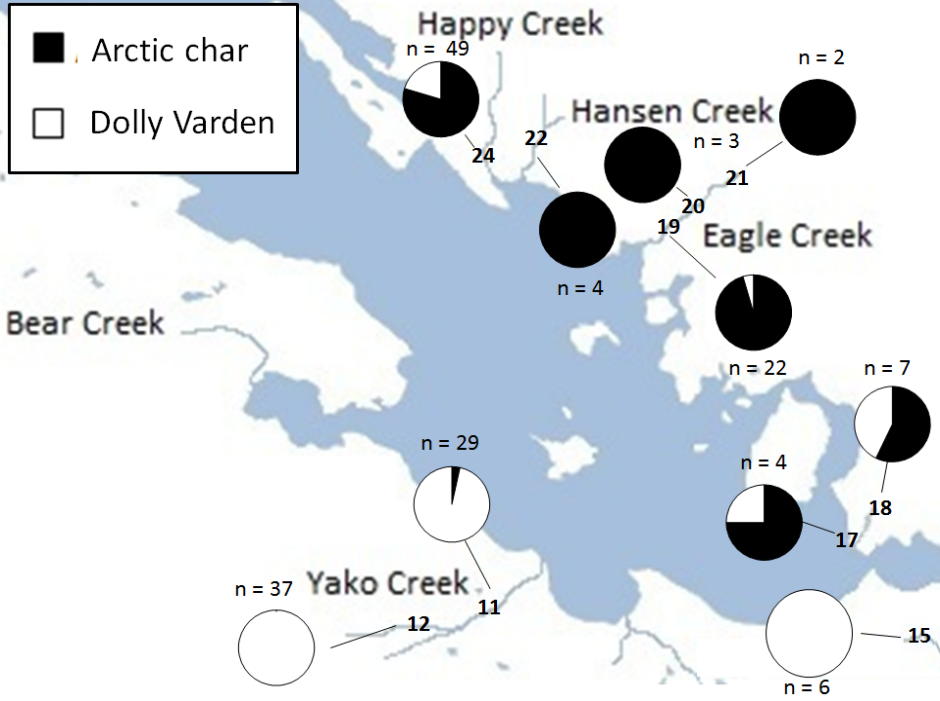


**Figure S6.** Proportion of genotypes defined by admixture (*Q*DV) values corresponding to Arctic char (*Salvelinus alpinus*) and Dolly Varden (*S. malma*) sampled from stream sites in Lake Aleknagik during summer 2013 and assayed at 12 microsatellite DNA loci. Arctic char were defined as having *Q*DV values of ≤ 0.21, Dolly Varden were defined as having *Q*DV ≥ 0.78, and hybrids were defined as having *Q*DV values between 0.21 and 0.78.


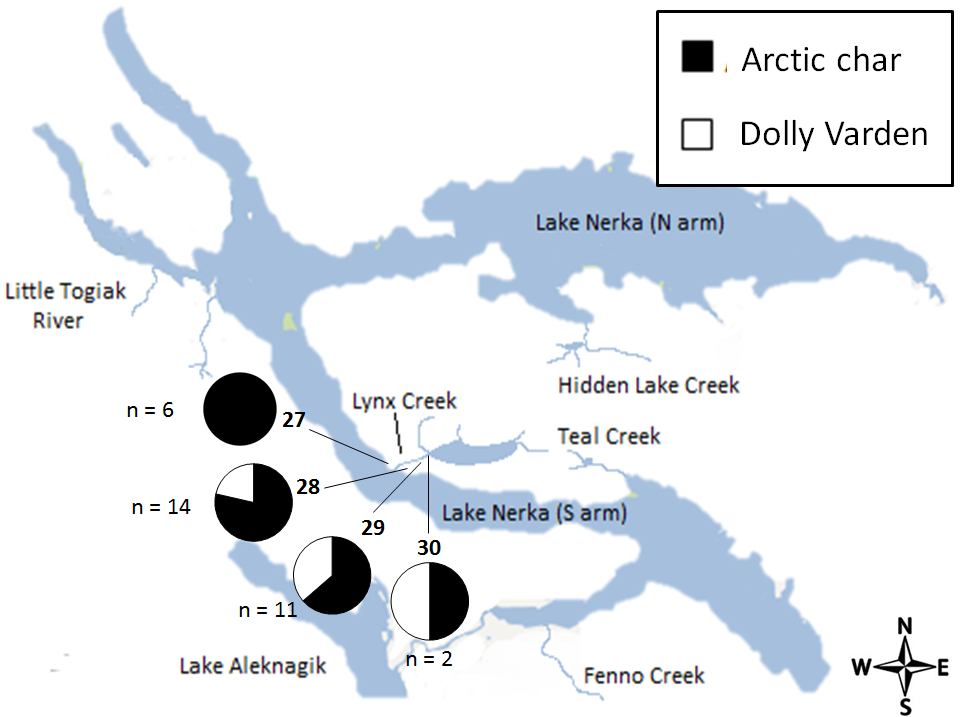


**Figure S7.** Proportion of genotypes defined by admixture (*Q*DV) values corresponding to Arctic char (*Salvelinus alpinus*) and Dolly Varden (*S. malma*) sampled from stream sites in Lake Nerka during summer 2013 and assayed at 12 microsatellite DNA loci. Arctic char were defined as having *Q*DV values of ≤ 0.21, Dolly Varden were defined as having *Q*DV ≥ 0.78, and hybrids were defined as having *Q*DV values between 0.21 and 0.78.
