# Supplementary material for: Low levels of hybridization between sympatric Arctic char (Salvelinus alpinus) and Dolly Varden char (Salvelinus malma) highlights their genetic distinctiveness and ecological segregation
Source: Ecol Evol. 2015 Jul 7;5(15):3031–45. doi: 10.1002/ece3.1583 (PMC4559047; doi:10.1002/ece3.1583)
Supplement: Supplementary file 2 [file ece30005-3031-sd2.docx]

**Table S1.** Genetic diversity values at 13 microsatelllite loci for Arctic char (*Salvelinus alpinus*) and Dolly Varden (*S. malma*) from two southwestern Alaskan Lakes with two sampling years (2012-2013) pooled. Sample codes: DV, Dolly Varden; AC, Arctic char; *N*, number of samples; *A*, number of alleles per locus; *A_R_*, Allelic richness; *H_O_*, observed heterozygosity; *H_E_*, expected heterozygosity; *F_IS_*, inbreeding coefficient of an individual relative to the subpopulation; *P* (*HWE*), probability of departure from Hardy-Weinburg equilibrium (*p*≤ 0.011).

| Locus |  | DV  (Aleknagik) | DV  (Nerka) | AC  (Aleknagik) | AC  (Nerka) |
| --- | --- | --- | --- | --- | --- |
| ***Sco220*** | *N* | 109 | 36 | 383 | 179 |
|  | *A* | 32 | 23 | 34 | 29 |
|  | *A_R_* | 3.7 | 5.2 | 33.8 | 28.8 |
|  | *H_O_* | 0.771 | 0.667 | 0.943 | 0.922 |
|  | *H_E_* | 0.939 | 0.944 | 0.95 | 0.937 |
|  | *F_IS_* | 0.18 | 0.297 | 0.008 | 0.016 |
|  | *P(HWE)* | 0* | 0* | 0.0327 | 0.119 |
| ***Sco200*** | *N* | 112 | 35 | 383 | 181 |
|  | *A* | 38 | 27 | 28 | 23 |
|  | *A_R_* | 3.7 | 5.3 | 27.3 | 22.7 |
|  | *H_O_* | 0.9375 | 0.943 | 0.898 | 0.873 |
|  | *H_E_* | 0.951 | 0.948 | 0.921 | 0.906 |
|  | *F_IS_* | 0.015 | 0.006 | 0.025 | 0.037 |
|  | *P(HWE)* | 0* | 0.0126* | 0.0297 | 0.108 |
| ***Smm22*** | *N* | 112 | 37 | 383 | 182 |
|  | *A* | 18 | 19 | 32 | 23 |
|  | *A_R_* | 3.4 | 5 | 31.4 | 22.9 |
|  | *H_O_* | 0.884 | 0.919 | 0.922 | 0.885 |
|  | *H_E_* | 0.886 | 0.924 | 0.916 | 0.934 |
|  | *F_IS_* | 0.002 | 0.06 | -0.006 | 0.053 |
|  | *P(HWE)* | 0.0089* | 0.305 | 0.175 | 0.0607 |
| ***Sco215*** | *N* | 2 | 3 | 381 | 171 |
|  | *A* | 3 | 3 | 5 | 6 |
|  | *A_R_* | 3 | 3 | 5 | 6 |
|  | *H_O_* | 0.5 | 0 | 0.276 | 0.222 |
|  | *H_E_* | 0.833 | 0.8 | 0.319 | 0.287 |
|  | *F_IS_* | 0.5 | 1 | 0.14 | 0.225 |
|  | *P(HWE)* | 0.333 | 0.0701 | 0.0374 | 0.0013* |
| ***Ssosl456*** | *N* | 111 | 37 | 381 | 183 |
|  | *A* | 8 | 4 | 7 | 6 |
|  | *A_R_* | 2.5 | 2.8 | 6.9 | 5.9 |
|  | *H_O_* | 0.784 | 0.703 | 0.617 | 0.623 |
|  | *H_E_* | 0.682 | 0.649 | 0.653 | 0.662 |
|  | *F_IS_* | -0.15 | -0.084 | 0.055 | 0.059 |
|  | *P(HWE)* | 0.0855 | 0.921 | 0.0214 | 0.389 |
| ***Otsg253b*** | *N* | 112 | 37 | 382 | 183 |
|  | *A* | 20 | 17 | 23 | 17 |
|  | *A_R_* | 3.3 | 4.7 | 22.6 | 17 |
|  | *H_O_* | 0.893 | 0.811 | 0.88 | 0.847 |
|  | *H_E_* | 0.861 | 0.891 | 0.907 | 0.901 |
|  | *F_IS_* | -0.038 | 0.091 | 0.031 | 0.06 |
|  | *P(HWE)* | 0* | 0.001* | 0.102 | 0* |
| ***Smm24*** | *N* | 101 | 36 | 348 | 183 |
|  | *A* | 25 | 21 | 21 | 21 |
|  | *A_R_* | 3.5 | 5.2 | 21 | 20.8 |
|  | *H_O_* | 0.871 | 0.944 | 0.905 | 0.907 |
|  | *H_E_* | 0.907 | 0.942 | 0.902 | 0.908 |
|  | *F_IS_* | 0.040 | -0.0030 | -0.0030 | -0.0050 |
|  | *P(HWE)* | 0* | 0.0218 | 0.658 | 0.987 |
| ***Omm1105*** | *N* | 108 | 37 | 383 | 182 |
|  | *A* | 18 | 15 | 11 | 12 |
|  | *A_R_* | 3.3 | 4.8 | 10.9 | 11.9 |
|  | *H_O_* | 0.694 | 0.833 | 0.773 | 0.747 |
|  | *H_E_* | 0.861 | 0.906 | 0.752 | 0.803 |
|  | *F_IS_* | 0.194 | 0.081 | -0.027 | 0.069 |
|  | *P(HWE)* | 0* | 0.242 | 0.311 | 0.369 |
| ***Otsg83b*** | *N* | 110 | 37 | 382 | 183 |
|  | *A* | 39 | 30 | 28 | 25 |
|  | *A_R_* | 3.7 | 5.45 | 27.5 | 24.6 |
|  | *H_O_* | 0.927 | 0.865 | 0.893 | 0.869 |
|  | *H_E_* | 0.951 | 0.958 | 0.911 | 0.917 |
|  | *F_IS_* | 0.026 | 0.101 | 0.02 | 0.052 |
|  | *P(HWE)* | 0* | 0.0144* | 0.113 | 0.177 |
| ***Smm17*** | *N* | 99 | 35 | 381 | 183 |
|  | *A* | 15 | 15 | 14 | 15 |
|  | *A_R_* | 3.3 | 4.5 | 13.7 | 14.9 |
|  | *H_O_* | 0.485 | 0.6 | 0.822 | 0.754 |
|  | *H_E_* | 0.857 | 0.88 | 0.856 | 0.851 |
|  | *F_IS_* | 0.436 | 0.321 | 0.04 | 0.114 |
|  | *P(HWE)* | 0* | 0* | 0.711 | 0.0882 |
| ***Sco216*** | *N* | 102 | 36 | 382 | 183 |
|  | *A* | 37 | 21 | 38 | 36 |
|  | *A_R_* | 3.7 | 5.26 | 37.6 | 35.8 |
|  | *H_O_* | 0.36 | 0.417 | 0.901 | 0.891 |
|  | *H_E_* | 0.943 | 0.946 | 0.958 | 0.962 |
|  | *F_IS_* | 0.616 | 0.563 | 0.06 | 0.075 |
|  | *P(HWE)* | 0.0043* | 0* | 0.028 | 0.0074* |
| ***Smm21*** | *N* | 108 | 35 | NA | NA |
|  | *A* | 6 | 4 | 1 | 1 |
|  | *A_R_* | 2.19 | 2.3 | 1 | 1 |
|  | *H_O_* | 0.574 | 0.429 | NA | NA |
|  | *H_E_* | 0.572 | 0.444 | NA | NA |
|  | *F_IS_* | -0.003 | 0.035 | NA | NA |
|  | *P(HWE)* | 0.0114* | 0.261 | NA | NA |
| ***Sco202*** | *N* | 107 | 35 | 374 | 180 |
|  | *A* | 7 | 3 | 17 | 14 |
|  | *A_R_* | 1.56 | 1.7 | 16.9 | 14 |
|  | *H_O_* | 0.28 | 0.314 | 0.797 | 0.806 |
|  | *H_E_* | 0.275 | 0.273 | 0.822 | 0.838 |
|  | *F_IS_* | -0.019 | -0.154 | 0.03 | 0.038 |
|  | *P(HWE)* | 0.157 | 1 | 0.439 | 0.0014* |

**Table S2.** Lake Aleknagik and Lake Nerka site ID numbers and names with corresponding sampling locale, year(s) sampled, sample size (*N*) and given proportions of samples classified as Arctic char (*Salvelinus alpinus*) (AC), Dolly Varden (*S. malma*) (DV) and hybrid (HYB). Arctic char were defined as having *Q*_DV_ values of ≤ 0.21, Dolly Varden were defined as having *Q*_DV_ ≥ 0.78, and hybrids were defined as having *Q*_DV_ values between 0.21 —0.78.

| **Site ID** | **Site Name** | **Locale** | **Year Sampled** | ***N*** | **AC/DV/HYB** |
| --- | --- | --- | --- | --- | --- |
| 1 | Youth Creek Mouth | Aleknagik | 2012 | 24 | 24/0/0 |
| 2 | Beach Seine 2S | Aleknagik | 2012 | 16 | 16/0/0 |
| 3 | Beach Seine 2N | Aleknagik | 2012 | 29 | 29/0/0 |
| 4 | Island off Hansen Creek | Aleknagik | 2012 | 18 | 18/0/0 |
| 5 | Bear Bay | Aleknagik | 2012 | 30 | 29/0/1 |
| 6 | Beach Seine 7S | Aleknagik | 2012 | 24 | 24/0/0 |
| 7 | Whitefish Bay | Aleknagik | 2012 | 20 | 20/0/0 |
| 8 | Bear Creek (lower) | Aleknagik | 2012 | 15 | 15/0/0 |
| 9 | Bear Creek (upper) | Aleknagik | 2012 | 3 | 1/2/0 |
| 10 | Yako Creek (lower) | Aleknagik | 2012 | 30 | 29/1/0 |
| 11 | Yako Creek (mid) | Aleknagik | 2013 | 29 | 1/28/0 |
| 12 | Yako Creek (upper) | Aleknagik | 2013 | 37 | 0/37/0 |
| 13 | Lower Whitefish Creek | Aleknagik | 2012 | 31 | 31/0/0 |
| 14 | Upper Whitefish Creek | Aleknagik | 2012 | 5 | 5/0/0 |
| 15 | Silversalmon Creek | Wood River | 2013 | 6 | 0/6/0 |
| 16 | Wood River | Wood River | 2012 | 8 | 0/7/1 |
| 17 | Mission Creek (lower) | Aleknagik | 2013 | 4 | 3/1/0 |
| 18 | Mission Creek (mid) | Aleknagik | 2013 | 7 | 4/3/0 |
| 19 | Eagle Creek (lower) | Aleknagik | 2012, 2013 | 36 | 35/1/0 |
| 20 | Eagle Creek (mid) | Aleknagik | 2012, 2013 | 7 | 5/2/0 |
| 21 | Eagle Creek (upper) | Aleknagik | 2012, 2013 | 14 | 13/1/0 |
| 22 | Hansen Creek (mid) | Nerka | 2012, 2013 | 21 | 17/4/0 |
| 23 | Hansen Creek (upper) | Nerka | 2012 | 4 | 3/1/0 |
| 24 | Happy Creek | Nerka | 2012, 2013 | 71 | 53/17/1 |
| 25 | Fenno Creek | Nerka | 2012 | 14 | 14/0/0 |
| 26 | Teal Creek | Nerka | 2012 | 20 | 18/2/0 |
| 27 | Lynx Creek (lower) | Nerka | 2012, 2013 | 17 | 16/1/0 |
| 28 | Lynx Creek (lower-mid) | Nerka | 2012, 2013 | 17 | 4/13/0 |
| 29 | Lynx Creek (mid-upper) | Nerka | 2012, 2013 | 17 | 13/4/0 |
| 30 | Lynx Creek (upper) | Nerka | 2012, 2013 | 63 | 44/18/1 |
| 31 | Little Togiak River | Nerka | 2012 | 18 | 18/0/0 |
| 32 | Hidden Lake Creek (lower) | Nerka | 2012 | 10 | 10/0/0 |
| 33 | Hidden Lake Creek (lower-mid) | Nerka | 2012 | 15 | 15/0/0 |
| 34 | Hidden Lake Creek (mid-upper) | Nerka | 2012 | 15 | 15/0/0 |
| 35 | Hidden Lake Creek (upper) | Nerka | 2012 | 18 | 18/0/0 |
|  |  |  |  |  |  |

**Table S3.** Summary of mean posterior probabilities with probability value ranges for individuals assigned with NEWHYBRIDS to each of six genotypic classes.

| Lake | Year | P_AC_ | P_DV_ | F1 | F2 | BX_AC_ | BX_DV_ |
| --- | --- | --- | --- | --- | --- | --- | --- |
| Aleknagik | 2012 | 0.994 (0.81-1.0) | 0.80 (0.51-0.93) | - | 0.81 (0.46-0.99) | - | 0.87 (0.51-0.99) |
| Aleknagik | 2013 | 0.995 (0.99-1.0) | 0.80 (0.47-0.99) | - | - | - | 0.93 (0.50-1.0) |
| Nerka | 2012 | 0.99 (0.82-1.0) | 0.82 (0.69-0.95) | - | - | - | 0.92 (0.51-0.99) |
| Nerka | 2013 | 0.99 (0.99-1.0) | 0.80 (0.60-0.99) | - | - | - | 0.73 (0.56-0.99) |

P, parental generation, F1 and F2, first and second filial generations respectively, BX, backcrossed, AC, Arctic char, DV, Dolly Varden
